# Narrative Review of High-Dose-Rate Interstitial Brachytherapy in Primary or Secondary Liver Tumors

**DOI:** 10.3389/fonc.2022.800920

**Published:** 2022-03-01

**Authors:** Efstratios Karagiannis, Iosif Strouthos, Agnes Leczynski, Nikolaos Zamboglou, Konstantinos Ferentinos

**Affiliations:** ^1^ Department of Radiation Oncology, German Oncology Center, Limassol, Cyprus; ^2^ Department of Medicine, School of Medicine, European University Cyprus, Nicosia, Cyprus

**Keywords:** liver cancer, liver metastases, HDR brachytherapy, liver brachytherapy, interstitial brachytherapy

## Abstract

The optimal management of intrahepatic malignancies involves a multidisciplinary approach. Although surgical resection has been considered the only curative approach, the use of several minimally invasive ablative techniques has dramatically increased the last two decades, mainly due to the fact that they provide similar oncological results with significantly decreased morbidity. Among these modalities, interstitial liver brachytherapy, probably the most flexible liver ablative method, with excellent clinical data on its safety and effectiveness, is frequently not even mentioned as an option in the current peer reviewed literature and guidelines. Brachytherapy is a type of radiotherapy utilizing radionuclides that are directly inserted into the tumor. Compared to external beam radiation therapy, brachytherapy has the potential to deliver an ablative radiation dose over a short period of time, with the advantage of a rapid dose fall-off, that allows for sparing of adjacent healthy tissue. For numerous malignancies such as skin, gynecological, breast, prostate, head and neck, bladder, liver and soft-tissue tumors, brachytherapy as a monotherapy or combined with external beam radiation therapy, has become a standard treatment for many decades. This review article aims to describe the high-dose-rate liver brachytherapy technique, its selection criteria, present its advantages and disadvantages, as well as the available clinical data, in order to help physicians to explore and hopefully introduce liver brachytherapy into their clinical routine.

## Introduction

With primary liver cancer being the seventh most commonly diagnosed cancer worldwide (1), while liver is the most common site of metastasis in patients with colorectal cancer (2), primary and secondary liver malignant tumors are frequently encountered. The best approach to optimize the management of these intrahepatic malignancies seems to be one that utilizes a core multidisciplinary liver tumor board, consisting of gastroenterologists/hepatologists, diagnostic and interventional radiologists, pathologists, surgical oncologists, medical oncologists, as well as radiation oncologists (3).

Although increasingly shifting, surgical approaches (transplantation or resection) remain the gold standard for patients with primary or secondary liver cancer. However, the majority of patients (80-90%) are poor surgical candidates due to age and comorbid medical conditions, functional status, severity of hepatic decompensation, unfavorable anatomical lesions location, insufficient future liver remnant, extent of metastases, history of extensive abdominal surgery or patient’s preferences (4–8). Against this background, over the last decades, several minimally invasive liver directed treatment modalities, including image-guided ablative and trans-arterial techniques (9), have been developed and are being used alone or in combination with traditional and newer systemic treatments, improving patient outcomes. As there is a whole armamentarium of such modalities (**Table 1**), it is crucial not only to choose the most appropriate but also the most suitable technique for each modality, tailoring the treatment concept to patient and tumor characteristics. Among these liver directed treatment modalities, interstitial brachytherapy (BT), probably the most flexible liver ablative method, with excellent available clinical data on its effectiveness and safety (10, 11), is frequently not even mentioned as an option in the current peer reviewed literature and guidelines. This article aims to describe the liver BT technique and its selection criteria, present its advantages and disadvantages, as well as the available clinical data in order to help the clinical decision-making process for patients needing a liver ablative treatment modality.

**Table 1:** Liver directed modalities for primary and secondary liver tumors.

Transarterial Techniques
Conventional trans-arterial chemoembolization (cTACE)
Chemotherapy bound to embolic particles in drug-eluting bead TACE (DEB-TACE)
Transarterial bland embolization (TAE)
Transarterial radioembolization (TARE)
Hepatic arterial infusion chemotherapy (HAI)
Ablation Techniques
Chemical Ablation
Percutaneous intralesional ethanol injection (PEI)
Percutaneous intralesional acetic acid injection (PAI)
Thermal Ablation
Radiofrequency ablation (RFA)
Microwave ablation (MWA)
Laser interstitial thermotherapy (LITT)
High intensity focused ultrasound (HIFU)
Cryosurgical ablation (CSA)
Other
Electrochemotherapy (ECT)
Irreversible electroporation (IRE)
Radiotherapy Ablation
Stereotactic Ablative Radiotherapy (SABR): photons, particles
Brachytherapy (BT): High-Dose-Rate, Low-Dose-Rate)

## Background

Although liver directed treatments have rapidly evolved the last decades, their efficacy or safety might be limited by tumor size, location, number of lesions, and/or amount of functional liver remnant (12–15), serious complications rate (14, 16), technique reproducibility and inconsistency (16, 17), or patient characteristics, such as implanted cardiac devices (18), cardiac arrhythmias (19), prior treatments and pathological lung shunt fraction (LSF) (20). Liver interstitial BT, and more specifically high-dose-rate (HDR) liver BT, as an alternative to the other liver-directed modalities has none of the above limitations. It is not limited by tumor size and localization or liver/tumor-specific density, blood perfusion rate, specific heat, electrical or thermal conductivity. Additionally, taking into account that larger radiation dose leads to greater cellular kill probability, the major advantage of BT lies in the physical dose distribution surrounding the radioactive source (most commonly Ir-192). While very high, precisely predicted radiation doses, able to sterilize all tumor cells, are delivered intra-tumoral, the steep dose fall-off, outside the tumor-target, accounts for greatly reduced doses to the surrounding healthy tissues. All the above can lead to excellent local disease control with minimal toxicity.

## History of Liver Brachytherapy

The concept of interstitial liver brachytherapy, as it is known today, was first described by Dritschilo et al. in 1986 (21). They have reported an ultrasound-guided, percutaneous placement of a 14 gauge closed-ended needle suitable for attachment to the Gamma Med II Ir-192 remote after-loader (Palo Alto, CA) in local anesthesia. The use of computed tomography (CT)-based treatment planning, as well as the prescribed dose of up to 50Gy to volumes up to 25cm^3^ in a single fraction, became major reference points for the evolution of liver brachytherapy. One year later, the same group (Georgetown group) reported their experience with intraoperative single fraction interstitial liver BT (22), while the Memorial Sloan Kettering group published the first low-dose-rate (LDR) liver BT results in 1990 (23). Although liver BT in Europe began in the late 1990s, the first systematic reports were published in the 2000s. Zamboglou, a believer in BT and a longtime BT pioneer, was the first to perform liver BT in Europe and the first to introduce CT-guidance for catheter insertion worldwide (24). The Offenbach group has reported the results of 31 patients with inoperable primary and secondary liver tumors, treated between 2000 and 2009 with CT-guided interstitial BT (10), while the Magdeburg group introduced a novel catheter implantation technique, which uses a trochar puncture needle, that is exchanged over a stiff angiographic guide wire for a flexible 6-F catheter sheath using Seldinger’s technique and the subsequent replacement of the wire by a closed-end 6F BT catheter, and reported their first results back in 2004 (25). It has to be noted that the extended clinical research conducted by Ricke et al. (Magdeburg Group), regarding multiple aspects of liver HDR BT, such as organs at risk (OARs) tolerance dose assessment (26–29), dose and fractionation evaluation (11, 30), magnetic resonance imaging (MRI) and positron emission tomography (PET) introduction in guidance and implantation accuracy, treatment planning, as well as tumor/tissue radiation response (29, 31–34) expanded the knowledge base of the whole brachytherapy community. The first experience regarding the feasibility of MRI-guided liver brachytherapy was published in 2001 by Kettenbach et al. (35).

## Patient Selection

As aforementioned, in the dynamic area of hepatic oncology, the value of a liver specific multidisciplinary board cannot be overstated. It is widely accepted that every patient should undergo formal evaluation by such a multidisciplinary tumor board. In general, liver HDR-BT patient selection criteria are similar to other ablation techniques, with BT being much more flexible in terms of location, size, and number of lesions. Heat or cold sink effects, proximity to major bile ducts are less of an issue for BT, if at all, than for thermal ablation techniques. Similarly, inter- or intra-fractional liver (and other OARs) motion, lesion size and location, as well as future liver reserve are less of a concern for BT compared to Stereotactic Ablative Radiation (SABR) (36).

Due to the lack of an international consensus defining (in)eligibility criteria for liver BT candidates, every institution has developed its own approach. Our inclusion/exclusion criteria are presented in **Table 2**.

**Table 2:** German Oncology Center (GOC) inclusion/exclusion criteria for HDR liver BT.

Inclusion Criteria	
Hepatobiliary Cancer	
Hepatocellular Carcinoma	Non-metastatic, unresectable tumor, transplant candidate (as bridge therapy)
	Non-metastatic, unresectable tumor(s), not transplant candidate (as definitive therapy), ≤ 4 liver lesions
	Non-metastatic tumor(s), non-surgical candidate (as definitive therapy), ≤ 4 liver lesions
	Oligometastatic disease (≤ 3 extrahepatic lesions amenable to locoregional treatment), ≤ 4 liver lesions in combination with systemic Tx.
	Recurrent/oligorecurrent/oligoprogressive intrahepatic disease (individualized concept)
Intrahepatic Cholangiocarcinoma	Non-metastatic, unresectable tumor(s), ≤ 4 liver lesions, in combination with systemic Tx
	Non-metastatic tumor(s), non-surgical candidate, ≤ 4 liver lesions, in combination with systemic Tx
	Oligometastatic disease (≤ 5 metastatic lesions amenable to locoregional treatment), ≤ 4 liver lesions in combination with systemic Tx.
	Recurrent/oligoreccurent intrahepatic disease (individualized concept)
Oligometastatic liver disease	≤ 4 liver lesions, with controlled extrahepatic disease or ≤ 3 extrahepatic lesions amenable to locoregional Tx in combination with systemic Tx
	Recurrent/oligorecurrent/oligoprogressive intrahepatic disease (individualized concept)
Exclusion Criteria	
ECOG 3, Child-Pugh Class C, Hb ≤ 8g/dl, Neutrophils ≤ 1500/mm^3^, Platelets ≤ 50000/mm^3^, INR ≥ 1.5, Bilirubin ≥ 5 μmol/dL	

## Technique

### Preplanning

Radiation dose delivery in HDR-BT, using the common after-loading technique, involves the positioning of non-radioactive, closed-ended catheters in the tumor-target and the subsequent loading of them with the radiation source (usually Ir-192). The source travels through the catheters and remains in predetermined positions (dwell positions) for a predetermined time (dwell time), in order to deliver the prescribed dose. As the possible dwell positions are physically constrained by the catheter path, accurate catheter positioning is essential.

Currently, pre-implant three-dimensional (3D) imaging is commonly used to manually determine the number and the position of the catheters. Virtual needles are usually placed to provide “optimal” dwell positions for a desired dose distribution, while maintaining a safe catheter access route. Although for small spherical lesions the pre-planning procedure is straightforward, for larger liver tumors and moreover in the proximity of OARs, defining the optimal number of catheters, their spatial configuration and the ideal spatiotemporal source stepping pattern within them, in the era of anatomy-based 3D planning, can be technically challenging (37). Unfortunately, computerized techniques based on mathematical optimization models, which can effectively solve these problems and provide superior implant quality, are not widely implemented in liver BT (38–40).

In our institution a deep expiration or inspiration breath-hold CT fused with pre-acquired liver specific MRI data is used for the inverse pre-planning procedure, followed by delineation of planning tumor volume (PTV), OARs and avoidance regions, including major vessels, gallbladder, bile ducts or dilated biliary channels. Virtual catheters, proposed by the hybrid inverse planning and optimization (HIPO) algorithm (40) for complex implants or manually determined for simpler implants, are placed, so that all selected dosimetric (PTV, OARs) and anatomical objectives (safe catheters pathway) are met. The dosimetric constraints used in published studies, as well as in our institution are described in **Table 3**. It has to be noted that in the majority of liver brachytherapy clinical studies more conservative dose constraints were used. We recommend using the tighter and more extensively studied constraints into clinical practice (**Table 3**). For cases where the dose to the stomach, duodenum, jejunum, ileum or colon is a limiting factor, balloon catheters can be used (48).

**Table 3:** German Oncology Center (GOC) dosimetric constraints for HDR liver BT.

Target/OARs	Constraints used in clinical studies	GOC Constraints	GOC Special considerations
PTV (Target)	D100% = PD (11, 25, 30, 41)	V100% > 95%	PD: 25Gy
Healthy liver tissue (Liver-GTVs)	V5Gy < 2/3 liver volume (11, 25, 30, 41, 42)	V10Gy < 2/3 liver volume and 700cc	For Re-irradiation*: V40Gy (ΣEQD_3Gy_2) < 2/3 liver volume and 700cc
	V10Gy < 2/3 liver volume (43)		
Esophagus/Stomach/Duodenum/Jejunum/Ileum	D1cc < 15Gy (11, 27, 30, 44, 45)	D1cc < 15Gy	For Re-irradiation*: D1cc < 85Gy( Σ EQD_3Gy_2)
	D1cc < 12Gy (46)		
	Dmax < 15Gy (47)		
	Dmax < 14Gy (42)		
Colon	D1cc < 15Gy (11, 30)	D1cc < 15Gy	
	Dmax < 18Gy (42)		
Spinal Cord	D1cc < 8Gy (11, 30)	D1cc < 14Gy	For Re-irradiation*: D1cc < 75Gy( Σ EQD_3Gy_2)
	Dmax < 15Gy (47)		
Kidneys (for GOC: individual and combined)	V7Gy < 2/3 kidney volume (43)	D1cc < 18Gy D200cc < 10Gy Dmean < 6Gy	For Re-irradiation*: D1cc < 90Gy(ΣEQD_3Gy_2)
			D200cc < 40Gy(ΣEQD_3Gy_2)
			Dmean < 16Gy(ΣEQD_3Gy_2)
Thoracic Wall	No constraint	D1cc < 23Gy	For Re-irradiation*: D1cc < 110Gy(ΣEQD_3Gy_2)
Great Vessels	No constraint	D1cc < 27Gy	For Re-irradiation*: D1cc < 240Gy(ΣEQD_3Gy_2)
Gallbladder	Dmax < 20Gy (42)	No constraint	Mandatory reporting of D1cc and Dmean

### Catheter Placement

Interstitial catheter implantation is usually performed under sedoanalgesia and local anesthesia enabling image guidance (US, CT, MRI). Breath hold techniques similar to pre-planning procedure are commonly used. Two similar catheter placement approaches have been described, with one incorporating the Seldinger technique (25) and one without this interventional radiology procedure step (10, 21). Regardless of implantation technique, the implant geometry should ideally replicate the virtual plan’s geometry, in order to achieve the user-selected dose distribution objectives. Upon completion of catheter placement, post-implant non-enhanced or contrast-enhanced CT/MRI images of the liver are acquired for the 3D treatment planning. Photo-documentation of the implant, catheter numbering and free-length (length of needles outside the patient) measurement, directly after the image acquisition, as well as patient transport to the treatment room, avoiding catheter displacement, is of great importance.

### Treatment Planning

CT-based planning is the treatment planning technique of choice, especially when TG-186 dose calculation algorithms are used (49). If TG-43 formalism and its updates are used (50–52), MRI-based planning is also feasible. Following the catheters reconstruction (digitization) and volumes of interest (VOIs) delineation, which include PTVs and OARS, a 3D-anatomy-based plan can be generated. In our institution a combined 3mm isotropic expansion of the gross tumor volumes (GTVs) to PTVs is used. Catheters reconstruction and VOIs delineation are simultaneously performed, which significantly reduces the treatment planning procedure time. The typical prescribed dose covering the 95% of the PTVs is 25Gy in a single fraction (**Table 3**). After the plan evaluation and the catheters free-length verification, the treatment can be delivered. Proton-pump inhibitors, serotonin 5-HT3 receptor antagonists and low-dose corticosteroids are administered routinely before treatment delivery.

### Clinical Results

With almost 20 years of clinical experience with HDR-BT of liver tumors, there is a large amount of published data. Most of them are chronologically presented in **Table 4**. Ricke et al. were the first to prospectively analyze the brachytherapy outcome of 36 secondary and two primary liver tumors ranging from 2.5 to 11 cm (mean 4.8 cm) in 37 patients (25). Twenty-one patients were treated with CT-guided HDR-BT alone, while sixteen patients received brachytherapy directly after MRI-guided laser-induced thermotherapy (LITT) due to suspected incomplete thermal ablation, mainly because of tumor size or location. They reported local control rates of 87% for the HDR-BT-group and 73% for the LITT followed by HDR-BT-group at 9 months and an OS rate of 69% at 12 months for all patients combined. One year later, Ricke et al. published the results of a prospective non-randomized phase II trial, which included 20 patients with an equal number of large (up to 10cm) or unfavorably located for thermal ablation, primary and secondary liver tumors (41). LC was 80% and 53% at 6 and 9 months respectively, while OS was 83% at 12 months. The same group explored the local tumor control in colorectal liver metastases after HDR-BT at various dose levels and managed to demonstrate a strong dependency between tumor control and dose escalation (29). The LC was 75% at 15 months for the whole cohort of patients and 95% if doses>25Gy were prescribed. Mohnike et al. treated 83 patients presented with 140 hepatocellular carcinomas (HCC), generating a LC rate of 95% and an OS rate of 64% at 12 months (30). The study by Schnapauff et al. investigated the clinical outcome of 15 patients with unresectable intrahepatic cholangiocarcinoma, treated with HDR-BT (53). After a median follow-up of 18 months, the median LC was 10 months and the median OS was 14 months after HDR-BT and 21 months after the initial diagnosis. Wieners et al. reported on 115 hepatic metastases of breast cancer in 41 patients (44). They were treated in 69 interventions using the most commonly used single fraction approach. LC was 97%, 93.5% and 93.5%, while OS was 97%, 79% and 60% at 6, 12 and 18 months respectively. Collettini et al. shared their experience treating 7 patients with 12 isolated ovarian cancer metastases to the liver, generating a LC and OS rate of 100% at 12months (59). The same group evaluated the clinical outcome of 35 patients with 19 large (5-7cm) and 16 very large (>7cm) HCC, as well as the outcome of 8 patients with 12 solitary gastro-esophageal adenocarcinoma metastases (54, 60). After a median follow-up of 8 and 12 months, the LC rate was 100% and 92.3%, for the patients with gastro-esophageal adenocarcinoma metastases and HCC respectively. Tselis et al. treated 41 patients with 50 unresectable primary and secondary liver tumors, reporting LC rates of 90%, 81% and 50% for primary and 89%, 73% and 63% for metastatic tumors at 6, 12 and 18 months respectively (57). The study by Collettini et al. analyzed the results of 80 patients with 179 unresectable colorectal liver metastases treated with liver BT in a single or multiple fractions. LC rates were 88.3%, 81.2% and 68.4% while OS rates were 87.6%, 57.3%, and 41.6% at 12, 24 and 36 months respectively (58). The same authors reported on 98 patients with 212 unresectable HCCs using a single fraction technique, generating LC rates of 91.5% at 21.1 months, as well as OS rates of 87.6%, 57.3% and 41.6% at 12, 24 and 36 months respectively (46). Denecke et al. explored the role of liver BT as bridging therapy for patients with HCC listed for liver transplantation, comparing it to trans-arterial chemoembolization (55). Their data show comparable or even better response and post-LT recurrence rates in favor of liver BT. Schippers et al. treated 27 patients with 52 neuroendocrine liver metastases in 40 sessions, reporting LC rates of 92%, 83% and 83%, as well as OS rates of 96%, 96% and 63% at 12, 36 and 60 months respectively (43). The study by Kieszko et al. evaluated the clinical outcome of 61 patients with 73 liver secondary tumors treated with HDR-BT. LC and OS rates were 88.7%, 70.7% and 96.7%, 79.6% at 6 and 12 months respectively (45). Omari et al. investigated the results of 14 patients with 54 renal cell liver metastases, treated with liver BT, reporting LC rate of 92.6% at 10.1 months and median OS of 51.2 months (62).

**Table 4:** Clinical data regarding HDR-BT of primary and secondary liver tumors.

Author	N	n	Entity	Size	Dose	Results	Complications
Mohnike et al. (30)	83	140	Primary (HCC)	5.8cm (1-15cm)	(15-25Gy)	12mo OS: 64%	7.2% major complications
	75	126		4.4cm (1-15cm)	mean: 17.6Gy (15-25Gy)	36mo OS: 25%	
						12mo LC 95%	
Schnapauff et al. (53)	15	15	Primary (CCC)	5.25cm (1-18cm)	median: 20Gy (15-20Gy)	Median LC: 10m	3.7% major complications
						Median OS: 14mo	
Collettini et al. (54)	35	35	Primary (HCC)	Mean: 7.1cm (5-12cm)	Median: 15Gy (15-20Gy)	12.5mo LC 92.3%	No complications
Collettini et al. (46)	98	212	Primary (HCC)	Mean: 5cm (1.8-12)	Mean: 16.51Gy (15-20Gy)	21.1mo LC 91.5%	0.47% major complications
						12mo OS 87.6%	0.47% minor complications
						24mo OS 57.3%	
						36mo OS 41.6%	
Denecke et al. (55)	12	12	Primary (HCC)	Mean: 3.6cm	Mean: 18.9Gy (15-25Gy)	12mo LC 90%	8.3% major complications
						36mo LC 90%	
Jonczyk et al. (56)	61	142	Primary (CCC)	Median: Subgroup A: 20.41ml (10-38ml)	Mean: 18.42Gy (12-20Gy)	6mo LC (A:98% B: 89%)	No complications
				Subgroup B: 69.25ml (40-148ml)		12mo LC (A: 87% B:78%)	
						24mo LC (A: 72% B:37%)	
						60mo LC (A: 72% B:37%)	
						6mo OS (A:94% B: 75%)	
						12mo OS (A: 68% B:63%)	
						24mo OS (A: 61% B:36%)	
						60mo OS (A: 36% B:12%)	
Ricke et al. (25)	37	38	Primary/Secondary	4.8cm (2.5-11cm)	mean: 17Gy (10-20Gy)	9mo LC 73-87%	5% major complications
						12mo OS 69%	41% minor complications
Ricke et al. (41)	20	20	Primary/Secondary	7.7cm (5.5-10.8cm)	mean: 17Gy (12-25Gy)	6mo LC 80%	10% major complications
						9mo LC 53%	40% minor complications
						12mo OS 83%	
Tselis et al. (57)	41	50	Primary/Secondary	Median: 84ml (38-1348ml)	Median: 20Gy (7-32Gy)	Primary: 6mo LC 90%	5% major complications
						12mo LC 81%	15.2% minor complications
						18mo LC 50%	
						Secondary:	
						6mo LC 89%	
						12mo LC 73%	
						18mo LC 63%	
Ricke et al. (11)	73	199	Colorectal metastases	5cm (1-13cm)	3 dose levels (15, 20, 25 Gy)	15mo LC 75% and 95% if PD > 25Gy	5% major complications
Collettini et al. (58)	80	179	Colorectal metastases	Mean: 2.85cm (0.8-10.7cm)	Mean: 19.1Gy (15-20Gy)	12mo LC 88.3%	No major complications
						24mo LC 81.2%	
						36mo LC 68.4%	
						12mo OS 87.6%	
						24mo OS 57.3%	
						36mo OS 41.6%	
Wieners et al. (44)	41	115	Breast-Ca metastases	Median: 4.4cm (1-11cm)	median: 18.5Gy (12-25Gy)	6mo LC 97%	1.4% major complications
						12mo LC 93.5%	8.6% minor complications
						18mo LC 93.5%	
						6mo OS 97%	
						12mo OS 79%	
						18mo OS 60%	
Collettini et al. (47)	37	80	Breast-Ca metastases	Mean: 2.5cm (0.8-7.4cm)	Mean: 18.57Gy (15-20Gy)	12mo LC: 90%	2.7% major complications
						12mo OS: 96%	
Collettini et al. (59)	7	12	Ovarian-Ca metastases	Mean: 3.19cm (1.3-12cm)	Median: 15Gy	12mo LC 100%	No complications
						12mo OS 100%	
Geisel et al. (60)	8	12	GEAC metastases	Median: 4.6cm (1.4-6.8cm)	Median: 20Gy (15-20Gy)	8.4mo LC 100%	11.1% minor complications
Schippers et al. (43)	27	52	NELM	Mean: 3.1cm (0.7cm-11cm)	Median: 20Gy (15-20+Gy)	12mo LC 92%	2.5% minor complications
						36mo LC 83%	
						60mo LC 83%	
						12mo OS 96%	
						36mo OS 96%	
						60mo OS 63%	
Geisel et al. (61)	10	16	RCC metastases	Median: 3.8cm (1-8.2cm)	Median: 20Gy	12mo LC: 90%	No major complications
						12mo OS: 100%	
Omari et al. (62)	14	54	RCC metastases	Mean: 2.9cm (0.7-13.9cm)	Median: 16.1Gy (6.5-27.4Gy)	10mo LC: 92.6%	No major complications
						Median OS: 51.2mo	16.2% minor complications
Wieners et al. (42)	20	49	Pancreas-Ca metastases	Mean: 2.9cm (1-7.3cm)	Mean: 18.1Gy (15-20Gy)	12mo LC: 91%	15% major complications
						12mo OS: 45%	
Drewes et al. (63)	16	45	Pancreas-Cametastases	Median: 2.2cm (1-11.2cm)	Median: 21Gy (5-29.1Gy)	Median PFS: 3.4mo	18% major complications
						Median OS: 8.9mo	
Kieszko et al. (45)	61	73	Secondary	Median: 42.9ml (2.7-174.9ml)	Mean D_90_:20.2Gy (15-25Gy)	6mo LC 88.7%	No major complications
						12mo LC 70.7%	5% minor complications
						6mo OS 96.7%	
						12mo OS 79.6%	
Kieszko et al. (45)	61	73	Secondary	Median: 42.9ml (2.7-174.9ml)	Mean D_90_:20.2Gy (15-25Gy)	6mo LC 88.7%	No major complications
						12mo LC 70.7%	5% minor complications
						6mo OS 96.7%	
						12mo OS 79.6%	

N, number of patients; n, number of lesions; HCC, hepatocellular carcinoma; CCC, cholangiocarcinoma; RCC, renal cell carcinoma; GEAC, gastro-esophageal adenocarcinoma; NELM, neuroendocrine liver metastases; LC, local control; OS, overall survival.

All the included published studies (**Table 3**) conclude that liver brachytherapy is a safe treatment approach, suggesting average major and minor complication rates below 4% and 6% respectively. Mohnike et al. conducted the largest known study, regarding liver brachytherapy complications and risk factors. They evaluated 192 patients and 343 interventions and reported major complication rate of 4.1%, equivalent to that reported for thermal ablation (64, 65). Major complications included hemorrhage grade III-IV (1.46%), ascites grade II (0.29%), ulcers (0.87%), radiation induced liver disease (0.5%), liver abscess (1.17%) and bile duct obstruction (0.29%), while minor complications included hemorrhage grade I (3.21%), ascites grade I (0.71%), pleural effusion grade I-II (12.2%) and pneumothorax grade I-II (1.75%). Pain and nausea were the most frequent patient reported symptoms (64).

## Discussion

Minimally invasive, image-guided ablation techniques are increasingly used for the treatment of primary or secondary liver malignancies, not only for patients that are poor or borderline surgical candidates but also for patients that prefer a minimally invasive procedure, with similar results. It should be noted, that although there are studies favoring surgery over ablation and vice versa (66, 67), these findings should be interpreted with caution, as currently, there are no results from randomized controlled trials (RCTs) available, directly comparing the outcome of an ablation method vs surgery. The eagerly awaited results of registered RCTs, such as the COLLISION (NCT03088150), the HELARC (NCT02886104) and the LAVA (ISRCTN52040363) trials, will hopefully enlighten this topic. Similarly, RCTs comparing available ablation methods, including liver HDR-BT, are needed.

Liver HDR-BT extends the therapeutic possibilities for hepatic lesions considering the limitations of more commonly used ablation techniques, as it is not limited by tumor size, location, number of lesions, and/or amount of functional liver remnant (12–15), serious complications rate (14, 16), technique reproducibility and inconsistency (16, 17) or patient characteristics such as implanted cardiac devices (18), cardiac arrhythmias (19), prior treatments and pathological lung shunt fraction (LSF) (20). The large amount of available clinical data suggests that liver HDR-BT is not only feasible and safe but also at least iso-effective or even superior to other ablation methods, considering that it generates similar oncologic outcomes, while most patients treated with liver HDR-BT were not appropriate candidates for other ablation techniques.

In the past, liver HDR-BT was almost exclusively offered as a palliative treatment with the aim of symptoms control and/or cytoreduction that could possibly delay the onset of further systemic therapies. Nowadays, with a better understanding of concepts such as oligometastasis or oligoprogression, liver HDR-BT can be used as a potentially curative option, alone or in combination with traditional or newer systemic therapies, including immunotherapy. Emerging data suggest that the highly heterogeneous and conformal dose distribution of brachytherapy, may be optimal for enhancing the immunogenic capacity of radiation at a tumor site while minimizing off-target antagonistic effects on peripheral immune cells, serving as a method of *in situ* tumor vaccination.

Despite the promising clinical results, the minimal (if any) limitations, the cost-effectiveness and the immunogenic potential of liver BT, it is frequently not even mentioned as an option in the current peer reviewed literature and guidelines. The European Society of Medical Oncology (ESMO) was the first and till now (to the best of the authors’ knowledge) the only society that includes liver HDR-BT as a treatment option in its clinical treatment guidelines for HCC and metastatic colorectal cancer. It is proven that all ablative techniques, when performed by experienced operators, can successfully treat primary or secondary liver tumors. At present, there is no ideal ablative technique that outperforms the rest. There are ablation techniques that may share technical aspects and are usually comparable, but each of them has unique advantages that make it the “optimal” technique in particular settings. In conclusion, the choice of the appropriate treatment method and technique should be multidisciplinary, based on patients’ and tumors’ characteristics, as well as the medical expertise provided by the given treatment center.

## Future Directions

Although liver-BT has dramatically advanced in the last two decades, its main challenge remains the level of expertise required for implementing it into clinical practice. A brief look at the published data shows that unfortunately only a few centers offer this valuable treatment modality as an option. The reason is definitely not the lack of evidence demonstrating patients’ benefit, but mostly the deficient education and training of the next generation of radiation oncologists, the real or perceived barrier of user dependency of BT, the lack of randomized trials including liver-BT, as well as reimbursement issues.

Regarding the education challenges, the brachytherapy and radiation oncology societies have to take action and carefully re-plan the radiation oncology residency programs, so that every resident has the opportunity to be exposed to the whole BT spectrum.

Eliminating the “user dependency” in BT is a more challenging step. With regard to liver-BT, widely accepted clinical practice guidelines for catheters-placement, tumor delineation, dose prescription, tolerance dose constraints and surveillance imaging modalities are absolutely required in order to minimize variations in treatment delivery and evaluation, making liver-BT more consistent and efficient. Additionally, the build of a worldwide accessible Common Liver BT Database, allowing to record anonymized clinical data would contribute to this direction. Closing the gap between what clinicians do and what scientific evidence supports, could be the solid base of multi-institutional randomized clinical trials allowing comparisons of liver-BT with other liver directed modalities. Finally, the clinical implementation of existing technologies such as electromagnetic tracking for catheter reconstruction, automatic applicator/templates digitization, 3d printing for custom applicators/templates design, or newer technologies, such as deep-learning methods for auto-segmentation, as well biological and radiobiological novelties such as diagnostic, predictive and prognostic biomarkers, is highly required.

Reimbursement is probably the most difficult issue to tackle. It is known that BT requires equipment, complex imaging, trained personnel and a lot of physician effort and reimburses so poorly, that centers offering it frequently incur a net financial loss. Healthcare providers have to understand that if clinical practices are unprofitable, they will fold, with major consequences for the patients but also for them in the long run.

## Conclusion

Image-guided liver HDR-BT is a well-studied, safe and effective treatment with minimal toxicity. Although not endorsed by the majority of oncological societies, it is probably the most flexible liver ablation technique, as it is not limited by patient or tumor characteristics, being a valuable adjunct in the large toolbox of interventional and irradiation techniques, for the treatment of primary and secondary liver cancers.

## Author Contributions

All authors have made substantial contributions to all of the following:

- the conception and design of the manuscript
- administrative support
- provision of study materials
- collection and assembly of the data
- data analysis and interpretation
- manuscript writing
- final approval of manuscript

## Conflict of Interest

The authors declare that the research was conducted in the absence of any commercial or financial relationships that could be construed as a potential conflict of interest.

## Publisher’s Note

All claims expressed in this article are solely those of the authors and do not necessarily represent those of their affiliated organizations, or those of the publisher, the editors and the reviewers. Any product that may be evaluated in this article, or claim that may be made by its manufacturer, is not guaranteed or endorsed by the publisher.
